# Chemically modified neoantigen-based immunotherapy for targeting KRAS^G12C^-driven tumors

**DOI:** 10.1016/j.tips.2023.02.004

**Published:** 2023-05

**Authors:** Mai Abdel Mouti, Siim Pauklin

**Affiliations:** 1 Botnar Research Centre, Nuffield Department of Orthopaedics, Rheumatology and Musculoskeletal Sciences, University of Oxford, Oxford OX3 7LD, UK

**Keywords:** resistance, KRAS^G12C^, targeted therapy, immunotherapy, neoantigens

## Abstract

The clinical efficacy and durability of KRAS^G12C^-targeted therapies are limited by the development of resistance mechanisms. Here, we provide a review of recent KRAS^G12C^-targeted therapy and immunotherapy-unifying strategies that utilize covalently modified peptide/MHC class I complexes as tumor-specific neoantigens to tag drug-resistant cancer cells for destruction with hapten-based immunotherapeutics.

## Resistance of KRAS^G12C^-driven tumors to targeted therapies

The small GTPase Kirsten rat sarcoma viral **oncogene** (see Glossary) homolog (*KRAS*) is the most frequently mutated member of the *RAS* gene family, and is considered the major oncogenic driver of highly lethal human malignancies. Among *KRAS* mutations, there is a high prevalence of mutations at G12 (81%), followed by G13 (14%), then Q61 (2%) amino acid residues, with five **missense mutations** including G12D, G12V, G12C, G13D, and Q61R accounting for the majority (70%) of all KRAS-mutant cases [1]. While compounds targeting G12V and G12D mutations are actively being investigated, clinical trials of chemical inhibitors targeting KRAS^G12C^, including sotorasib (AMG 5110) and adagrasib (MRTX849), have demonstrated promising anti-cancer activity, with acceptable tolerance [2,3].

There is, however, a substantial proportion of patients who show short-term responses to KRAS^G12C^ inhibitors due to the development of drug resistance [4., 5., 6.]. Diverse genomic aberrations are involved in the evasion of KRAS^G12C^-targeted therapies. These include, for instance, acquired *KRAS* alterations (G12D/R/V/W, G13D, Q61H, R68S, H95D/Q/R, and Y96C); high-level amplification of the *KRAS*^*G12C*^ **allele**; *MET* amplification; activating mutations in *NRAS*, *BRAF*, *MAP2K1,* and *RET*; oncogenic fusions involving *ALK*, *RET*, *BRAF*, *RAF1*, and *FGFR3*; loss-of-function mutations in *NF1* and *PTEN* [4]; and polyclonal alteration-mediated rebound activation of the RAS–MAPK signaling pathway [5]. In addition, the histological transformation from adenocarcinoma to squamous cell carcinoma as well as epithelial-to-mesenchymal transition induced by PI3K activation have been identified as mechanisms of resistance to direct inhibition of oncogenic KRAS^G12C^ [4,7].

Therefore, it is essential to develop novel therapeutic strategies that can overcome such resistance mechanisms in order to enhance the clinical efficacy and durability of KRAS^G12C^ inhibitors.

## Neoantigen-based immunotherapy for targeting KRAS^G12C^-mutant cancer cells

Neoantigens are modified peptides that are generated by a variety of mechanisms, including genomic mutations, altered transcriptomic variants, post-translational modifications, as well as viral infections. They are recognized by the immune system as non-self/foreign antigens, and are thus capable of eliciting an antitumor immune response when specifically recognized by T cells [8]. In clinical trials, neoantigen-based antitumor immunotherapies have demonstrated promising efficacy and safety in multiple cancer subtypes [9]. The unique expression of neoantigens by tumor cells renders neoantigen-based immunotherapy a promising tumor-specific therapeutic approach that poses a minimal risk of off-target effects [10]. Here, we discuss innovative approaches that utilize **haptenated** peptide/**MHC** class I complexes as tumor-specific neoantigens to specifically tag drug-resistant, KRAS^G12C^-mutant cancer cells, and trigger antitumor immune responses when recognized by hapten-based immunotherapeutics.

## Immunotherapeutics targeting ARS1620-derived cancer neoantigens

**Figure 1:**
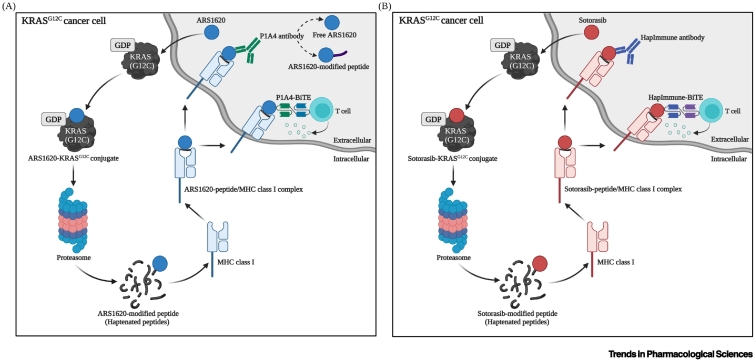
Hapten-based immunotherapeutic approaches for targeting drug-resistant, KRAS^G12C^-mutant cancer cells. In both (A) Zhang *et al*.’s and (B) Hattori *et al*.’s approaches, a covalent drug inhibitor enters the cell and forms an irreversible, covalent bond with cysteine 12 residue of inactive GDP-bound mutant KRAS^G12C^, which locks the protein in its inactive conformation. Upon proteasomal degradation of the drug-KRAS^G12C^ conjugate, haptenated peptide fragments are generated which are then incorporated into a compatible MHC class I molecule. The drug-peptide/MHC class I complex is presented on the cell surface, where they act as recognition elements for P1A4 or HapImmune antibodies. Bispecific T cell engagers (BiTEs) constructed from P1A4 and HapImmune antibodies induce a cytotoxic T cell response against drug-resistant, KRAS^G12C^-mutant cancer cells, leading to cell death.

A recent study conducted by Zhang *et al*. demonstrated the possibility of targeting drug-resistant, KRAS^G12C^-mutant cancer cells with hapten-based immunotherapeutics [11]. The authors utilized the covalent KRAS^G12C^ inhibitor ARS1620 to create tumor-specific neoantigens that are amenable to targeting by immunotherapy. The proteasomal degradation of ARS1620–KRAS^G12C^ conjugate results in the generation of peptide fragments that are bound to the drug inhibitor (haptenated peptides). These are then presented on the cancer cell surface as they incorporate into a compatible MHC class I molecule, forming a drug-modified peptide/MHC class I complex that serves as a target for antibody recognition of cancer cells (Figure 1A).

Recombinant antibodies have been successfully used to target tumor-specific MHC class I epitopes [12]. Zhang *et al*. identified five antibody clones, including the P1A4 clone which was studied in more detail, whose major antigenic determinant is ARS1620. This resulted in a lack of distinction between ARS1620-modified peptide, ARS1620-modified peptide/MHC class I complex, and free ARS1620, rendering further evaluation in preclinical models inconvenient due to high levels of free ARS1620 in the circulation [11]. In order to overcome this limitation, Zhang *et al*. generated an additional antibody that was more selective for the drug-modified peptide/MHC class I complex over the drug-modified peptide alone, and was also less sensitive to free drug competition. However, further studies are needed to evaluate its therapeutic potential and safety profile. Nevertheless, **bispecific T cell engagers (BiTEs)** constructed from the P1A4 clone induced a cytotoxic T cell response against KRAS^G12C^-mutant cancer cells, including those resistant to ARS1620, leading to cell death.

## Immunotherapeutics targeting sotorasib-derived cancer neoantigens

In another attempt to target drug-resistant, KRAS^G12C^-mutant cancer cells, Hattori and colleagues demonstrated a similar therapeutic strategy to Zhang *et al*., but using different approaches. Hattori *et al*. utilized sotorasib as the covalent KRAS^G12C^ inhibitor, and developed different types of antibodies, referred to as HapImmune antibodies, to target drug-modified peptide/MHC class I complexes in KRAS^G12C^-mutant cancer cells (Figure 1B) [13].

The neoantigen-based immunotherapeutic platforms demonstrated by Zhang *et al*. [11] and Hattori *et al*. [13] take advantage of the unique expression of oncogenic KRAS^G12C^ mutation by tumor cells [1], to selectively elicit an immune response against drug-resistant, KRAS^G12C^-mutant cancer cells when covalently modified with a KRAS^G12C^ inhibitor, as well as the retention of drug inhibitor-KRAS^G12C^ conjugation by drug-resistant tumors [4]. They both heavily rely on the covalent modification of mutant KRAS^G12C^ by KRAS^G12C^ inhibitors, which results in the subsequent display of haptenated peptides on the cancer cell surface. This facilitates the immune system’s recognition of mutant oncoproteins, which would normally be intracellular. Both approaches have the potential to integrate a broad repertoire of covalent drug inhibitors, regardless of whether the drug inhibitor is pharmacologically active or not, allowing them to target a wide variety of drug-resistant tumors. In addition, BiTEs constructed from HapImmune antibodies [13], as well as P1A4 [11], induce cytotoxic T cell responses against drug-resistant, KRAS^G12C^-mutant cancer cells (Figure 1).

While the study conducted by Zhang *et al*. primarily focused on studying ARS1620–KRAS^G12C^ [11], Hattori *et al*. proved that their approach could be extended to other drug inhibitor–target pairs besides sotorasib–KRAS^G12C^, which included **osimertinib**–epidermal growth factor receptor and **ibrutinib**–Bruton’s tyrosine kinase. HapImmune antibodies are poly-specific for multiple **human leukocyte antigen (HLA)**-A allele–hapten complexes, suggesting loosening of MHC restriction. However, they do not bind to the free drug [13], allowing concomitant administration of drug inhibitors and HapImmune antibodies. By contrast, P1A4 antibody, developed by Zhang *et al*., does not distinguish between the drug-modified peptide, drug-modified peptide/MHC class I complex, or the free drug inhibitor [11], which limits its therapeutic applicability.

## Concluding remarks

Emerging preclinical and clinical data demonstrate the limited clinical efficacy and durability of KRAS^G12C^ inhibitors due to the development of drug resistance mechanisms. The innovative covalently modified neoantigen-based immunotherapeutic approaches, recently published by Zhang *et al*. and Hattori *et al*., combine the benefits of targeted therapy with immunotherapy to kill drug-resistant, KRAS^G12C^-mutant cancer cells, which renders evasion mechanisms to targeted therapies more challenging. In addition, the flexibility of both approaches, in terms of formability and properties of covalent drug inhibitors, provides a wider range of treatment options for various types of tumors that are resistant to targeted therapies, as well as an opportunity to target more tumor-specific neoantigens therapeutically.

It is, however, necessary to examine both approaches in preclinical and clinical settings to assess their safety and validate their therapeutic efficacy against KRAS^G12C^, as well as other targets. Further studies are also required to determine if acquired resistance mechanisms may occur, including secondary gene mutations that interfere with the ability of the drug inhibitor to bind to its target protein, or if prolonged administration of therapeutic antibodies can lead to autoimmune diseases, or if some covalent drug inhibitors may have off-target effects due to their nonspecificity. These additional investigations will enable us to form a more comprehensive view of how these approaches could advance cancer immunotherapy.

## Glossary

**Allele**: one of two or more versions of DNA sequence at a given genomic location.
**Bispecific T cell engager (BiTE)**: engineered monoclonal antibodies that form a link between cancer cells and T cells.
**Hapten**: small molecule that induces antibody production only when conjugated to a larger molecule.
**Human leukocyte antigen (HLA)**: cluster of genes located on the short arm of chromosome 6 that encodes various cell surface markers, antigen-presenting molecules, and other proteins involved in regulating functions of the immune system. They are simply the human version of the MHC complex.
**Ibrutinib**: small-molecule inhibitor of Bruton’s tyrosine kinase.
**MHC**: recognition elements located on the cell surface of all nucleated cells and platelets. They mainly present peptide fragments generated from the proteasomal degradation of intracellular proteins to cytotoxic T cells.
**Missense mutation**: a change in DNA that encodes for a different amino acid residue at a particular position within the resulting protein.
**Oncogene**: a gene that leads to cancer development when it is mutated or expressed at a high level within the cell.
**Osimertinib**: third-generation irreversible epidermal growth factor receptor tyrosine kinase inhibitor.

## References

[1] Prior I.A. (2020). The frequency of Ras mutations in Cancer. Cancer Res..

[2] Hong D.S. (2020). KRAS(G12C) inhibition with sotorasib in advanced solid tumors. N. Engl. J. Med..

[3] Ou S.I. (2022). First-in-human phase I/IB dose-finding study of adagrasib (MRTX849) in patients with advanced *KRAS*^G12C^ solid tumors (KRYSTAL-1). J. Clin. Oncol..

[4] Awad M.M. (2021). Acquired resistance to KRAS^G12C^ inhibition in cancer. N. Engl. J. Med..

[5] Tanaka N. (2021). Clinical acquired resistance to KRAS^G12C^ inhibition through a novel KRAS switch-II pocket mutation and polyclonal alterations converging on RAS-MAPK reactivation. Cancer Discov..

[6] Zhao Y. (2021). Diverse alterations associated with resistance to KRAS(G12C) inhibition. Nature.

[7] Adachi Y. (2020). Epithelial-to-mesenchymal transition is a cause of both intrinsic and acquired resistance to KRAS G12C inhibitor in KRAS G12C-mutant non-small cell lung cancer. Clin. Cancer Res..

[8] Xie N. (2023). Neoantigens: promising targets for cancer therapy. Signal Transduct. Target Ther..

[9] Blass E. (2021). Advances in the development of personalized neoantigen-based therapeutic cancer vaccines. Nat. Rev. Clin. Oncol..

[10] Kishton R.J. (2020). Strength in numbers: identifying neoantigen targets for cancer immunotherapy. Cell.

[11] Zhang Z. (2022). A covalent inhibitor of K-Ras(G12C) induces MHC class I presentation of haptenated peptide neoepitopes targetable by immunotherapy. Cancer Cell.

[12] Hsiue E.H. (2021). Targeting a neoantigen derived from a common *TP53* mutation. Science.

[13] Hattori T. (2022). Creating MHC-restricted neoantigens with covalent inhibitors that can be targeted by immune therapy. Cancer Discov..

